# Addressing social risk factors in the inpatient setting: Initial findings from a screening and referral pilot at an urban safety-net academic medical center in Virginia, USA

**DOI:** 10.1016/j.pmedr.2022.101935

**Published:** 2022-07-28

**Authors:** Askar Chukmaitov, Bassam Dahman, Sheryl L. Garland, Alan Dow, Pamela L. Parsons, Kevin A. Harris, Vanessa B. Sheppard

**Affiliations:** a Virginia Commonwealth University (VCU) School of Medicine, Department of Health Behavior and Policy, 830 E. Main Str, Richmond, VA 23219, USA; b VCU Health System, Richmond, USA; c VCU School of Medicine, Division of Hospital Medicine; VCU Health Sciences for Interprofessional Education & Collaborative Care; VCU Health Continuing Education; VCU Department of Health Administration, Richmond, USA; d VCU School of Nursing, Department of Family and Community Health Nursing; Richmond Memorial Health Foundation, Richmond, USA; e VCU School of Medicine Dean's Office for Diversity, Equity and Inclusion, Richmond, USA; f VCU Massey Cancer Center, Richmond, USA

**Keywords:** Social determinants of health, Social needs, Social risk factors, Screening, Patient characteristics, Safety-net hospital

## Abstract

Social Determinants of Health (SDOH) impact health outcomes; thus, a pilot to screen for important SDOH domains (food, housing, and transportation) and address social needs in hospitalized patients was implemented in an urban safety-net academic medical center. This study describes the pilot implementation and examines patient characteristics associated with SDOH-related needs. An internal medicine unit was designated as a pilot site. Outreach workers approached eligible patients (n = 1,135) to complete the SDOH screening survey at time of admission with 54% (n = 615) completing the survey between May 2019 and July 2020. Data from patient screening survey and electronic health records were linked to allow for examination of associations between SDOH needs for food, housing, and transportation and various demographic and clinical characteristics of patients in multivariate logistic regression models. Of 615 screened patients, 45% screened positive for any need. Of 275 patients with needs, 33% reported needs in 2, and 34% – in 3 domains. Medicaid beneficiaries were more likely than patients with private health insurance to screen positive for 2 and 3 needs; Black patients were more likely than White patients to screen positive for 1 and 3 needs; Patients with no designated primary care physician status screened positive for 1 need; Patients with a history of substance use disorder screened positive for all 3 needs. SDOH screening assisted in addressing social risk factors of inpatients, informed their discharge plans and linkage to community resources. SDOH screening demonstrated significant correlations of positive screens with race/ethnicity, insurance type, and certain clinical characteristics.

## 1 Introduction

Social determinants of health (SDOH) are defined broadly as contextual factors that are exogenous to the healthcare system, but influence health outcomes and health status of populations. (Centers for Disease Control and Prevention., 2018) Commonly referred to as social needs, SDOH include fundamental domains such as income, education, food, housing, and transportation, among others. (Centers for Disease Control and Prevention., 2018, American Hospital Association, 2017) Social needs are associated with higher rates of emergency department utilization, hospital readmissions, and mortality risk. (Calvillo-King et al., 2013, Emechebe et al., 2019, Spatz et al., 2020) Effective SDOH screening is crucial for identification of patients with social needs and is therefore integral to quality improvement efforts addressing SDOH-related needs in health care settings. (Andermann, 2018).

A growing majority of health care stakeholders has promoted the integration of SDOH screening into broader health care settings. (Chisolm et al., 2019, Daniel et al., 2018, O’Toole et al., 2016, Thornton and Persaud, 2018) However, most studies examining the use of SDOH screening are limited to ambulatory care, emergency department, and pediatric patient populations. (Fritz et al., 2020, Gottlieb et al., 2014, Gottlieb et al., 2016, Weinreich et al., 2016) Despite significant progress in recognizing the importance of SDOH-informed care delivery, over 70% of hospitals in the U.S. do not screen patients for key social needs^15^ and many screening hospitals do not actively address patients’ social risks^4^ even as hospitalization may be a time when interventions to address social needs may have a positive impact on health outcomes. (Fraze et al., 2019) In addition, inpatient SDOH screening may provide valuable insight into patient care, improve care processes, and facilitate better planning of post-discharge care through referrals and linkage of patients to appropriate community-based resources. Conversely, a failure to adequately screen for and address patient social needs may contribute to excess utilization of emergency and inpatient services and worsen existing disparities in post-discharge outcomes4, (Spatz et al., 2020, Greysen et al., 2013).

Virginia Commonwealth University (VCU) Health, an urban safety-net academic medical center in Richmond, Virginia, serves a patient population burdened by high levels of social need. The City of Richmond faces pervasive racial/ethnic and economic disparities, represented by well-documented gaps in food security, housing stability, and transportation issues. (Blueprint, 2018, Central Virginia Legal Aid Society, 2019) These social needs and risk factors likely contribute to pronounced health disparities that are exemplified by a 20-year gap in life expectancy between neighboring census tracts in the city of Richmond. (VCU Center on Society and Health, 2014, County Health Rankings, 2021).

Prevalent social needs and the safety-net designation of VCU Health provide unique opportunities for collaboration with community-based partners to implement SDOH-related initiatives in the greater Richmond area. One of these initiatives is an inpatient SDOH screening and referral pilot aimed at addressing the food insecurity, housing instability, and transportation issues in an inpatient setting. This study describes the pilot implementation and community engagement efforts, reports the frequency of patients screening positive for social needs in an internal medicine unit, and examines associations of patient characteristics with the levels of social needs in screened patients. A deeper understanding of demographic, socioeconomic, and clinical characteristics of patients who screen positive for social needs may provide important insights for developing new programs and policies to address negative SDOH in inpatient settings.

## 2 Methods

### 2.1 Implementation of SDOH screening and referral pilot and community engagement

As part of the VCU Strategic Plan and Health Equity Initiative, VCU, VCU Health System, and community partners agreed to systematically address key negative SDOH – food insecurity, housing instability, and transportation issues – actionable through community-engaged interventions in education, patient services, and research. The SDOH screening and referral pilot was part of the initiative to address key negative SDOH in hospitalized patients.

Because hospital readmissions and inpatient mortality have been linked to negative SDOH, (Calvillo-King et al., 2013) an internal medicine unit with a readmission rate above the hospital-wide average was designated as the pilot site. The initiative provided all unit staff with training in SDOH on the importance of staff confidence, knowledge, and resource recognition to the successful integration of SDOH-related services into health care delivery. (Thornton and Persaud, 2018, County Health Rankings, 2021) All staff attended the SDOH training, which resulted in significant improvements to SDOH-related knowledge, attitudes, and practices. (Chukmaitov et al., 2020) Outreach workers, recruited VCU’s public health students, were also trained on a comprehensive screening tool, assessment of SDOH, identifying related needs, and prompting patients regarding referral or linkage to community-based resources.

The screening piloted in this study was modeled after the Health Leads: a widely used SDOH screening tool that is publicly available online and based on recommendations for SDOH screening among diverse patient populations. (Henrikson et al., 2019, The Health Leads Screening Toolkit, 2018) Self-reported food, housing, and transportation needs were the primary screening targets with additional open-ended prompts for any other social needs.

**Fig. 1:**
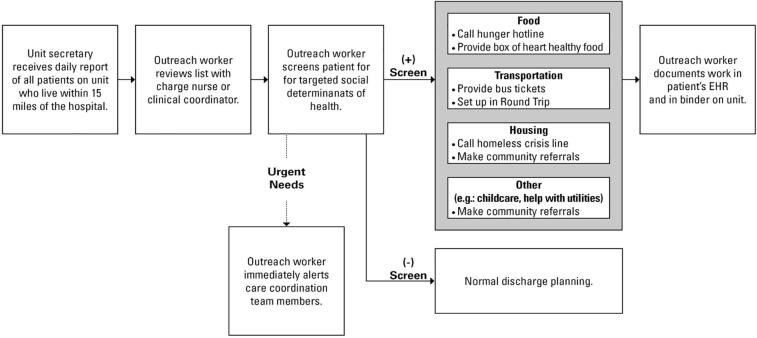
VCU Health, North 5, SDOH Screening and Referral Pilot Model.

Patients who screened positive for any social need were offered appropriate resources or referred to an external community partner for assistance at discharge. Screening results were recorded in EHR as free text, incorporated in daily interdisciplinary rounds (where multidisciplinary teams reviewed patient information displayed on a wide screen monitor in a conference room and discussed patients’ health status), and used to inform patients’ discharge plans. A food pantry at VCU Health was established and supplied by Feed More Feed More (2021), the main hunger relief agency in the region^25^ and provided a week’s worth supply of heart healthy food to address immediate food needs in patients at discharge. In addition, patients with food needs were connected with regional food pantries and referred to a hunger hotline for Supplemental Nutrition Assistance Program, Meals on Wheels enrollment assistance, and other long-term solutions. Moreover, VCU Health initiated closed loop tracking with Feed More and coordinated with the Feed More’s representatives to determine regional pantry utilization of discharged patients with food insecurity. Patients with housing need were referred to a hotline for a local housing crisis center and provided resource guides with income-based housing information. Transportation needs were addressed by providing Medicaid transportation support through RoundTrip rides Roundtrip (2021) and bus tickets redeemable to the Greater Richmond Transit Company – greater Richmond’s public transit system. VCU Health’s care coordination team and outreach workers identified urgent key social risks (e.g., currently being homeless or having no food) and other risk factors (e.g., exposure to violence, issues with unpaid utility bills) as barriers for safe discharge and assisted patients as part of the pilot activities and their normal job duties (Fig. 1).”.

### 2.2 Study population

A cross-sectional study design was used to determine the SDOH needs among adult patients (≥18 years of age) living in the VCU Health catchment area who were admitted to the internal medicine pilot site between May 2019 and July 2020. Patients with mental health conditions such as cognitive impairment that diminished their ability to participate in the survey were excluded. In addition, patients residing outside of the VCU Health’s catchment area and the community partners’ reach were excluded from the study, as it was difficult to follow up with them in the post discharge period. Outreach workers then approached all eligible patients (n = 1,135), introduced themselves, defined SDOH, and described the purpose of the screening for quality improvement. Patients who declined to participate in the screening were thanked and marked as non-participants. Outreach workers have electronically completed the SDOH screening survey with a 54% (n = 615) response rate.

Data from screening survey and electronic health records (EHR) were linked to allow for examination of associations between social need and various demographic and clinical characteristics of screened patients. These characteristics were also compared for total screened patients (n = 615) and those who declined screening (n = 520) to allow for understanding of characteristics which may be associated with participation in the SDOH screening pilot.

### 2.3 Measures

Responses from SDOH screening survey items were compiled to define three SDOH needs for food, housing, and transportation. In addition to developing individual dichotomous need variables, a 4-level need domain parity variable was created. (Andermann, 2018, Cole and Nguyen, 2020, Page-Reeves et al., 2016) This variable indicates if patients screened positive for social needs in 0, 1, 2, or all 3 of the addressed SDOH domains (food, housing, transportation) and was used as the dependent variable in the logistic regression models.

Patient demographic characteristics included age at the time of screening or index visit for those who refused screening, sex (female or male), race/ethnicity as a proxy for influence of racism (non-Hispanic White, non-Hispanic Black, or other race/ethnicity), (Flanagin et al., 2021 Aug 17) insurance coverage (private, Medicaid, Medicare, or other), and high-risk neighborhood. The high-risk neighborhood indicator variable was generated using patient zip codes and according to Richmond city subdivisions (districts) which have been identified as facing disproportionately high social need and low life expectancy. (VCU Center on Society and Health, 2014) Hispanic/Latino, Asian, Native American, and multiracial patients were underrepresented in the sample and were therefore included in the other race/ethnicity category. Uninsured patients were similarly underrepresented in the sample due to Medicaid Expansion in Virginia in 2019 and were included in the other insurance category. Patient primary care status was measured by an indicator variable representing patients with and without a designated primary care physician (PCP) at time of screening. Key comorbid conditions were extracted from the EHR using the International Classification of Diseases-tenth version (ICD10) and represented by indicator variables for diabetes, cardiovascular disease (CVD), substance use disorder (SUD), mental illness, and sepsis/septicemia. A dichotomous variable to indicate a high case-mix index (a weighted composite score of patient case severity) was developed for patients whose indices were above the sample mean relative to those with indices below the mean.

### 2.4 Statistical analysis

Descriptive analyses utilizing chi-square tests were performed to compare SDOH needs across patient subgroups. Mutually-exclusive need-specific groups were generated by cross-tabulating patients who reported any food, any housing, or any transportation need, allowing for descriptive examination of patients by their unique single- or multi-need profiles. Multivariate logistic regression models were developed to examine the association between SDOH needs and patient characteristics when controlling for a range of relevant covariates and confounders. A sensitivity analysis evaluated the Coronavirus Disease 2019 (COVID-19) effects on the SDOH need domains for hospitalized patients in pre- vs. post-COVID periods. A categorical variable of hospital admissions before and after March 2020 was added to the multivariate regressions. No statistically significant differences in pre- vs. post-COVID periods were found and thus not reported in the models. All analyses were performed using SAS 9.4. (SAS, 2021) This study protocol was approved by the institutional review board of Virginia Commonwealth University.

## 3 Results

### 3.1 Descriptive findings

Among all screened patients (n = 615), 45% screened positive for at least one SDOH need (n = 275). Relative to screened patients with no SDOH needs, patients who screened positive for a SDOH need were significantly more likely to fall within the 45–64 age bracket (55.3% vs. 41.2%, p < 0.0001), be Black (72.0% vs. 64.1%, p < 0.0001) or other race/ethnicity (7.3% vs. 1.9%, p < 0.0001), be covered by Medicaid (42.6% vs. 19.1%, p < 0.0001), have a history of substance use (47.6% vs. 30.6%, p < 0.0001) or mental illness (13.8% vs. 7.9%, p < 0.05) (Table 1). Relative to all screened patients (n = 615), patients who declined screening (n = 520) were statistically similar across nearly all characteristics, with the exceptions of race/ethnicity (p < 0.05), history of substance use (p < 0.05), or sepsis/septicemia (p < 0.01) (Table 1).

**Table 1:** Screening Participation & SDOH Need by Patient Characteristics.

	ANY SDOH Need	No SDOH Need	TOTAL SCREENED	Declined/Not Screened	TOTAL SAMPLE
	(n = 275)	(n = 340)	(n = 615)	(n = 520)	(n = 1,135)
Gender	p = 0.715		p = 0.175		
Male	47.6% (131)	49.1% (167)	48.5% (298)	52.5% (273)	50.3% (571)
Female	52.4% (144)	50.9% (173)	51.5% (317)	47.5% (247)	49.7% (564)

Age Group	p < 0.001**		p = 0.221		
Ages 18–45	22.6% (62)	20.6% (70)	21.5% (130)	21.2% (110)	21.3% (242)
46–64	55.3% (152)	41.2% (140)	47.5% (292)	42.3% (220)	45.1% (512)
65–74	14.2% (39)	21.8% (74)	18.4% (113)	20.8% (108)	19.5% (221)
75 and older	8.0% (22)	16.4% (56)	12.6% (78)	15.8% (82)	14.1% (160)

Race/Ethnicity	p < 0.0001**		p = 0.026*!		
White	20.7% (57)	34.7% (118)	28.5% (175)	32.5% (169)	30.3% (344)
Non-Hispanic Black	72.0% (198)	64.1% (218)	67.6% (416)	61.0% (317)	64.6% (733)
Other Race/Ethnicity	7.3% (20)	1.9% (4)	3.9% (24)	6.5% (34)	5.1% (58)

Insurance	p < 0.0001**		p = 0.221		
Private	12.4% (34)	20.0% (68)	16.6% (102)	14.4% (75)	15.6% (177)
Medicaid	42.6% (117)	19.1% (65)	29.6% (182)	27.9% (145)	28.8% (327)
Medicare	40.4% (111)	55.9% (190)	48.9% (301)	50.2% (261)	49.5% (562)
Other Payer	4.7% (13)	5.0% (17)	4.9% (30)	7.5% (39)	6.1% (69)

High-Risk Neighborhood	p = 0.961		p = 0.296		
Yes	51.3% (141)	51.5% (175)	51.4% (316)	48.3% (251)	50.0% (5637)
No	48.7% (134)	48.615% (165)	48.6% (299)	51.7% (269)	50.0% (568)

Designated PCP	p = 0.229		p = 0.313		
Yes	78.2% (215)	82.1% (279)	80.3% (494)	77.9% (405)	79.2% (899)
No	21.8% (60)	17.9% (61)	19.7% (121)	22.1% (115)	20.8% (236)

Substance Use Disorder	p < 0.0001**		p = 0.053		
Yes	47.6% (131)	30.6% (104)	38.2% (235)	32.7% (170)	35.7% (405)
No	52.4% (144)	69.4% (236)	61.8% (380)	67.3% (350)	64.3% (730)

Cardiac Disease	p = 0.119		p = 0.460		
Yes	26.6% (73)	21.2% (72)	23.6% (145)	21.7% (113)	22.7% (258)
No	73.4% (202)	78.8% (268)	76.4% (470)	78.3% (407)	77.3% (877)

Diabetes	p = 0.183		p = 0.433		
Yes	13.5% (37)	10.0% (34)	11.5% (71)	13.1% (68)	12.3% (139)
No	86.5% (238)	90.0% (306)	88.5% (544)	86.9% (452)	87.8% (996)

Mental Health	p = 0.018**		p = 0.676		
Yes	13.8% (38)	7.9% (27)	10.6% (65)	11.4% (59)	10.9% (124)
No	86.2% (237)	92.1% (313)	89.4% (550)	88.6% (461)	89.1% (1011)

Sepsis/Septicemia	p = 0.725		p = 0.002*!		
Yes	8.7% (24)	7.9% (27)	8.3% (51)	14.0% (73)	10.9% (124)
No	91.3% (251)	92.1% (313)	91.7% (564)	86.0% (447)	89.1% (1011)

Case Mix Index	p = 0.683		p = 0.327		
High	22.5% (62)	21.2% (72)	21.8% (134)	19.4% (101)	20.7% (235)
Low	77.5% (213)	78.8% (268)	78.2% (481)	80.6% (419)	79.3% (900)

**: p-value of overall chi-square tests between SDOH and no SDOH needs < 0.05; *! p-value of overall chi-square tests between total screened groups and Declined/Not Screened < 0.0.5.

**Fig. 2:**
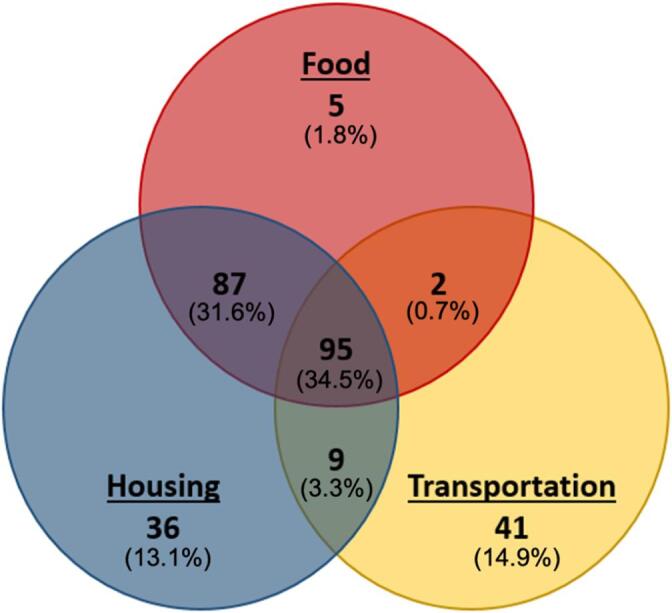
Patients who Screened Positive by Need (n = 275).

Of need-positive patients, one-third (n = 93) reported 2 needs, while a little more than one-third (n = 95; 35%) reported needs in all 3 domains. Fig. 2 displays the frequency of each SODH need and the overlap among and between each need. Among the 275 patients who screened positive for any domain of SDOH need, 36 (13%) screened positive for housing need only, 41 (15%) for transportation need only, and 5 (2%) for food need only. Eighty-seven patients (32%) screened positive for both food and housing need, representing the largest dual-need group. Patients screening positive for all 3 SDOH needs represented the largest group overall, with 95 patients (35%) having all three needs (food, housing, transportation).

### 3.2 Multivariate findings

Black patients were more than twice as likely as non-Hispanic White patients to screen positive for one SDOH need (OR 2.22, 95% CI 1.17, 4.19) and nearly twice as likely to screen positive for all 3 needs (OR 1.85, 95% CI 1.02, 3.36) (Table 2). Medicaid beneficiaries were more likely than the privately insured to screen positive for 2- and 3- needs [2 needs (OR 2.51, 95% CI 1.20, 5.28) and 3 needs (OR 5.37, 95% CI 2.31, 12.49)]. Patients with no designated PCP were nearly twice as likely to screen positive for 1 need (OR 2.05, 95% CI 1.07, 3.92). Patients with a history of SUD were over 3 times as likely as those with no SUD history to screen positive for all three domains of need (OR 3.195, 95% CI 1.85, 5.53). However, patients with a history of SUD were not statistically significantly associated with screening positive for one or two social needs. Moreover, patients with comorbid cardiac diseases, diabetes, mental health disorders, and sepsis/septicemia, and those with high case-mix index were not associated with screening positive for any domains of need.

**Table 2:** Multinomial Regression Results – SDOH Need Level by Patient Characteristic.

		1 SDOH Need	2 SDOH Needs	3 SDOH Needs
Gender	Male	ref	ref	ref
	Female	1.294 (0.75, 2.231)	1.332 (0.808, 2.194)	1.235 (0.738, 2.067)

Age Group	18–45	ref	ref	ref
	46–64	1.885 (0.893, 3.977)	1.189 (0.64, 2.21)	1.328 (0.7, 2.518)
	65–74	1.885 (0.701, 5.07)	0.641 (0.255, 1.613)	0.84 (0.322, 2.193)
	75 and older	0.891 (0.269, 2.951)	0.588 (0.211, 1.643)	0.621 (0.186, 2.072)

Race/Ethnicity^^^	White	ref	ref	ref
	Non-Hispanic Black	2.216** (1.17, 4.194)	1.55 (0.888, 2.718)	1.848* (1.017, 3.36)

Insurance	Private	ref	ref	ref
	Medicaid	2.17 (0.994, 4.743)	2.388** (1.135, 5.025)	5.23** (2.24, 12.195)
	Medicare	0.926 (0.407, 2.11)	1.331 (0.616, 2.877)	1.993 (0.8, 4.967)
	Other Payer	0.651 (0.125, 3.382)	1.494 (0.408, 5.464)	1.66 (0.371, 7.415)

High-Risk Neighborhood	Yes	0.681 (0.396, 1.172)	0.926 (0.565, 1.518)	0.825 (0.495, 1.376)
	No	ref	ref	ref

Designated PCP	Yes	ref	ref	ref
	No	2.049* (1.07, 3.922)	0.834 (0.415, 1.673)	1.339 (0.704, 2.547)

Substance Use Disorder	Yes	0.873 (0.48, 1.587)	1.296 (0.765, 2.195)	3.195** (1.85, 5.53)
	No	ref	ref	ref

Comorbidities	Cardiac Disease	1.008 (0.494, 2.056)	1.423 (0.784, 2.58)	1.375 (0.743, 2.544)
	No Cardiac Disease	ref	ref	ref

	Diabetes	1.94 (0.832, 4.527)	0.852 (0.379, 1.918)	0.936 (0.405, 2.164)
	No Diabetes	ref	ref	ref

	Mental Health	0.396 (0.111, 1.412)	1.799 (0.872, 3.711)	1.283 (0.592, 2.777)
	No Mental Health	ref	ref	ref

	Sepsis/Septicemia	1.015 (0.38, 2.708)	0.576 (0.187, 1.772)	2.163 (0.945, 4.953)
	No Sepsis/Septicemia	ref	ref	ref

Case Mix Index	High	0.994 (0.511, 1.931)	1.098 (0.593, 2.032)	1.321 (0.71, 2.46)
	Low	ref	ref	ref

*p < 0.05; **p < 0.01; Male, age 18–45, white, privately insured, Other Zip Code, had a PCP, no SUD history, no cardiac disease, no diabetes, no mental health history, no sepsis, and low case mix were the reference groups. ^^^Patients in the Other race/ethnicity group were excluded from regression analysis due to small screening (n = 24).

## 4 Discussion

The pilot demonstrated a large proportion of positive screens for social needs. Forty five percent (45%) of patients screened positive for at least one social need. Among those with a social need, 33% screened positive for 2 needs and 34% screened positive for needs in all 3 domains. Consistent with our findings, a study conducted in a primary care setting found that 46% of patients screened positive for at least 1 area of social need, while 63% of those had multiple needs. (Fusaro et al., 2018) Housing instability and food insecurity emerged as the most commonly reported social needs among the inpatient population, also consistent with priority needs identified by communities in the greater Richmond area. (Central Virginia Legal Aid Society, 2019, County Health Rankings, 2021) Collectively, these findings suggests that the inpatient population of an academic medical center may be similar in SDOH-related needs to the non-hospitalized populations though the hospitalized population may be in a more fragile life situation, needing a greater support in the inpatient setting, on-site access to food pantry, and linkages to community-based resources at discharge.

Significant differences in positive screens across patient subgroups by race/ethnicity, insurance type, and clinical characteristics were detected. We found that Black patients were over twice as likely to screen positive for 1 need and nearly twice as likely to screen positive 3 needs when compared to non-Hispanic White patients. National estimates show that Black patients are 1.6 to 2.7 times more likely to experience food insecurity and up to 3.5 times more likely to experience homelessness over the lifetime than White patients. (Gupta et al., 2020, Schanzenbach and Pitts, 2020, Jacobs, 2011) The racial gaps in positive screens likely contribute to the utilization and outcome disparities, which underscore the importance of the SDOH screening and addressing social needs for systematically disadvantaged populations. (Kushel et al., 2006, LaVeist et al., 2011, Syed et al., 2013, Gadson et al., 2017) Similarly, Medicaid beneficiaries were more likely to screen positive at both the 2 and 3 need parity levels, reflecting rates of social need previously reported by Medicaid beneficiaries in other settings. (Chisolm et al., 2019, Walker et al., 2016, Alley et al., 2016) While race/ethnicity and insurance status may indicate individuals who are at higher or lower risk for having SDOH needs, these measures are imprecise alone and careful screening appears to be a superior approach to identifying individuals at risk rather than relying solely on administrative or demographic data. Thus, incorporating SDOH screening results into EHR offers important insight into patient care that can improve care processes and hospital discharge planning.

Screening, however, should result in assistance for patients with identified social risks. For example, a growing number of state Medicaid agencies provide housing and habitation services, nutrition education, transportation, and other services to Medicaid beneficiaries with social needs. (Bachrach and Guyer, 2016, Meyers et al., 2010) These services are often coordinated via innovative delivery and payment models, including Accountable Care Organizations and the Patient-centered Medical Homes. (Baicker et al., 2013) The demonstrated role of these services within broader initiatives to achieve value-based care show promise for improving quality, access, and utilization among Medicaid beneficiaries. (Spencer et al., 2015, AHC, 2020) More recently, CMS launched an initiative to develop Accountable Health Communities to address SDOH and improve population health in at-risk communities. (National Academies of Sciences, Engineering, and Medicine et al., 2019) Additionally, SDOH initiatives are contingent on health care workers’ ability to identify at risk patients and link them to appropriate resources, organizational capabilities (e.g., outreach workers) to integrate SDOH screening into care processes and evaluate effectiveness of the SDOH interventions (i.e., data analytics), as well as external resources (e.g., community-based organizations) to coordinate patients’ social services in the post-discharge period. (Hefner et al., 2015) To date, only limited evidence exists on the effectiveness of addressing patients’ social risk factors in health care delivery settings. Our future research will evaluate the effectiveness of health care workers’ SDOH training, utilization of social services by at risk patients, and the SDOH pilot’s impact on patient outcomes (e.g., emergency department visits and hospital readmissions). However, more research is needed to better understand how best to integrate social services into health care delivery to achieve tangible improvements of patient outcomes, i.e. whether direct partnerships of health care providers and community-based organizations, new payment and delivery care models, or a combination of various organizational forms, payment models, government and community-based social services are most effective for addressing patients’ negative SDOH and improving health outcomes.^47^.

While patients with no designated PCP were nearly twice as likely to screen positively for one SDOH need in this study, the causality of this association should be more closely examined. Social determinants, such as transportation issues, may act as barriers to PCP access. (Cole and Nguyen, 2020, Owens et al., 2020) At-risk patients with no PCP access may require a higher level of care coordination in the post-discharge period. Potentially, SDOH needs and lack of a PCP may be self-reinforcing barriers that increase the change of hospital readmission and poor health outcomes multiplicatively.

Although most patient comorbidities and case-mix index were not associated with SDOH needs, we discovered that patients with a history of SUD were over 3 times more likely to screen positive for needs in all 3 domains, suggesting a high co-occurrence of social risks in this population as consistent with prior findings. (Furness et al., 2004) This relationship was notably absent at the 1- and 2- need levels. Similar to our findings, a recent study of national claims data reports that regions with higher rates of opioid-related inpatient and emergency department visits are disproportionately urban, low-income, and racially-segregated with a lower-proportion of Hispanic/Latino residents. (Henderson et al., 2008) Urban safety net providers should be aware of barriers shown to impact delivery of standard care for patients with SUD history and needs in food, housing, and other social services (McNeely et al., 2018, McGinnis et al., 2018) This population may need a robust support that includes both addressing SDOH needs and providing addiction services. The lack of association between SDOH needs and other diagnoses and case-mix index is also an important finding that should steer people away from a disease- or diagnosis-specific approach to interventions beyond the group of patients with SUDs.

## 5 Limitations

This study has limitations. The pilot was implemented in a single academic medical center and the findings may be unique to VCU Health and nearby communities. Nevertheless, VCU Health is a large urban safety-net hospital, and the findings of our study may be of interest to other academic safety net providers serving urban areas with large proportions of underserved or vulnerable populations. Some patients declined to participate in the SDOH screening and no information was collected on reasons why patients decided to forgo the survey; however, a relatively high response rate was achieved (56%) and patient characteristics for non-respondents were similar to respondents across nearly all characteristics. In addition, because we relied on administrative and billing codes for some data, these sources may have included errors common for secondary data sources.

## 6 Conclusion

This study’s recommendation to expand SDOH screening to inpatient settings, addressing food insecurity and providing transportation and timely referrals to community-based housing resources, is consistent with general guidance from leading stakeholders. (Bachrach and Guyer, 2016, Meyers et al., 2010, Byhoff et al., 2017) Physicians, nurses, faculty, administrators, and other allied health care workers all have a critical role to play in addressing patients’ negative SDOH. In this study, VCU Health prioritized SDOH screening to begin to address patients’ social risk factors. VCU Health’s faculty devoted their time and expertise to develop the SDOH training for health care workers on SDOH. In addition, being an academic medical center has allowed for hiring and training of highly motivated public health students as outreach workers. Moreover, VCU Health’s administrative staff has developed mutually beneficial relationships with leaders of local community-based organizations who offered resources and coordinated social care services to at risk patients. Having an analytical capacity to conduct the SDOH survey, collect and analyze data, was important for evaluating the SDOH pilot’s effectiveness. Finally, VCU Health and community partners were able to leverage the initial SDOH pilot’s success to obtain extramural funding for expansion of the SDOH-related initiatives.

This study demonstrates how certain approaches to the SDOH screening and referral interventions can be structured to meet the needs across a diverse population of hospitalized patients. Health care workers needed appropriate training and organizational buy-in to address commonly reported barriers to SDOH screening in inpatient settings and build capacity for sustainable resource linkage.(Thornton and Persaud, 2018) It was also important to merge and analyze the SDOH survey and EHR data, as significant correlations of patients’ social risk factors with race/ethnicity, insurance type, and certain clinical characteristics were identified. The SDOH screening results that are incorporated into clinical care can inform patient discharge plans, linking patients who screen positive for social needs to community-based resources and tracking resource utilization in a post-discharge period. As such, with appropriate organizational support and community engagement, SDOH screening and referral to community-based resources can be implemented in urban safety-net academic medical centers that serve inpatient populations of high social need. Moreover, urban safety-net academic medical centers may benefit from implementing screening approaches in inpatient settings and exploring new service delivery options and initiatives that show promise in addressing SDOH-related risks and improving patient outcomes.

## Declaration of Competing Interest

The authors declare that they have no known competing financial interests or personal relationships that could have appeared to influence the work reported in this paper.

## Data Availability

The authors do not have permission to share data.
